# Supplementary material for: Confined-microtubule assembly shapes three-dimensional cell wall structures in xylem vessels
Source: Nat Commun. 2023 Nov 13;14:6987. doi: 10.1038/s41467-023-42487-w (PMC10643555; doi:10.1038/s41467-023-42487-w)
Supplement: Supplementary file 1 — Supplementary Information [file 41467_2023_42487_MOESM1_ESM.pdf]

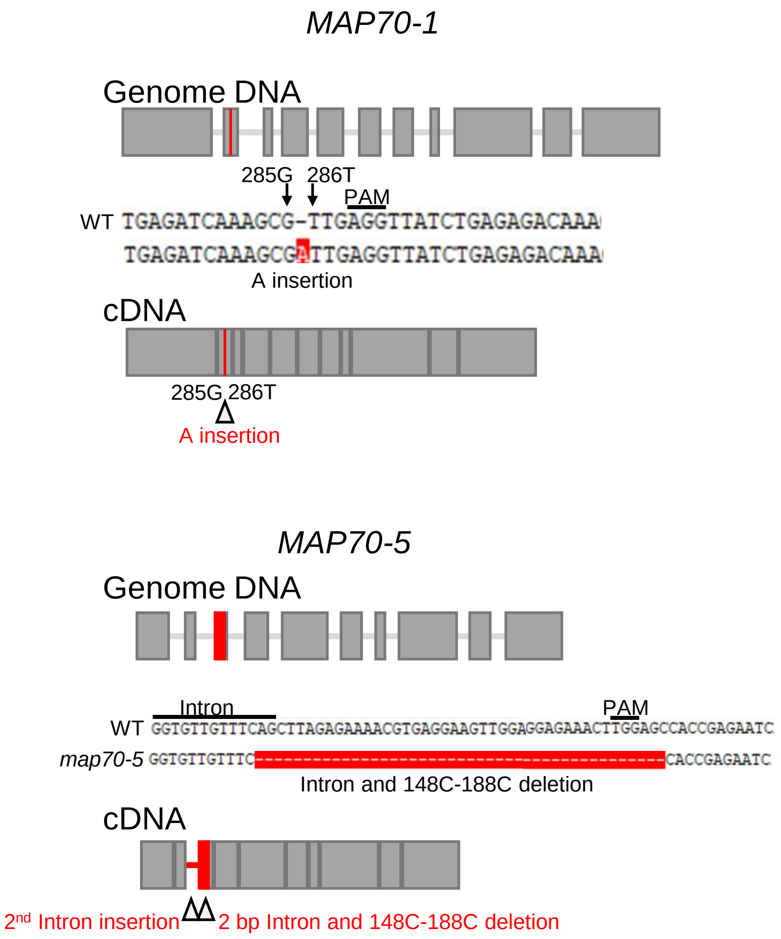

Supplemental Figure 1. Mutation sites in genomic DNA and cDNA of *map70-1* and *map70-5*

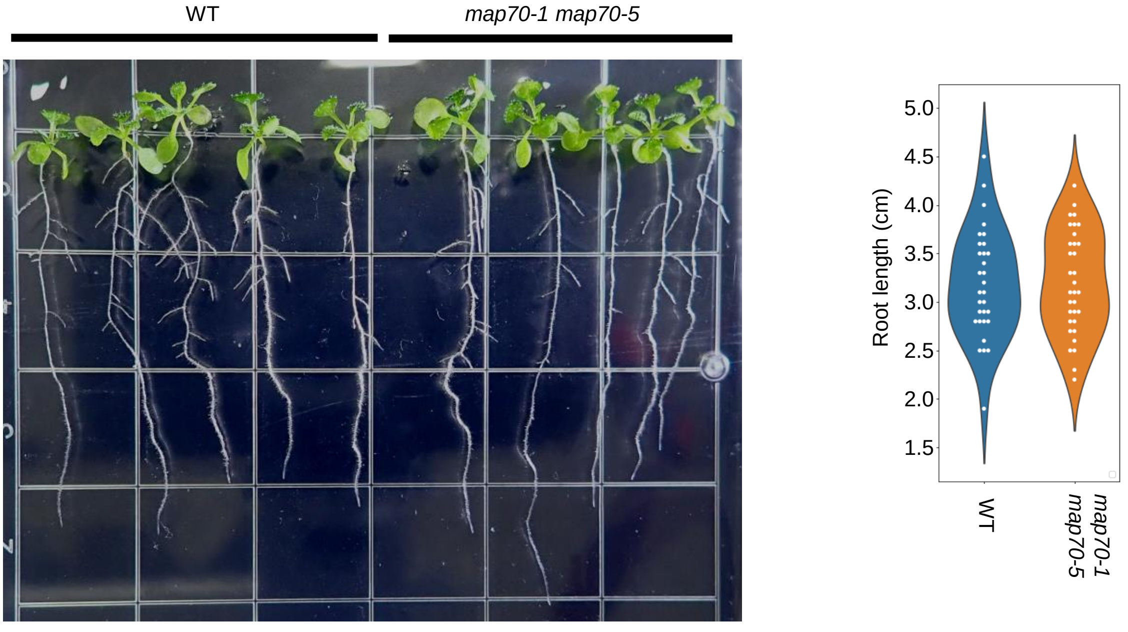

Supplemental Figure 2. ***map70-1 map70-5*** **plants grow normally**
(A) 10-days-old seedlings of wild type (WT) and *map70-1 map70-5* plants.
(B) Length of primary roots of wild type (WT) and *map70-1 map70-5* plants.
N = 31 (WT) and 33 (*map70-1 map70-5*) plants.

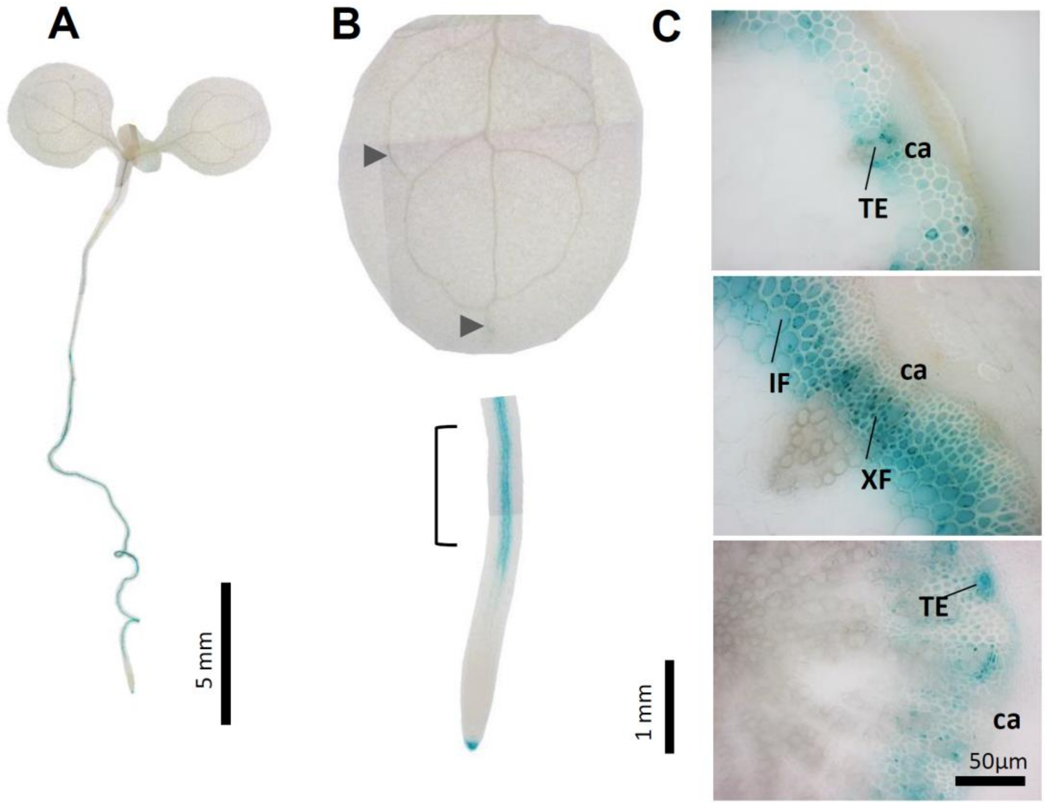

Supplemental Figure 3. ***MAP70-5*** **promoter is specific to xylem tissues**
(A) 7-day-old whole seedling of *pMAP70-5:GUS* showing β-glucuronidase activity in living tracheary elements (TEs).
(B) Close-ups of cotyledon and root highlighting β-glucuronidase activity in living TEs.
(C) 5-week-old floral stem of *pMAP70-5:GUS* showing β-glucuronidase activity in xylem tissues including TEs, xylary fibers (XF) and interfascicular fibers (IF). Note that ca indicates cambium.

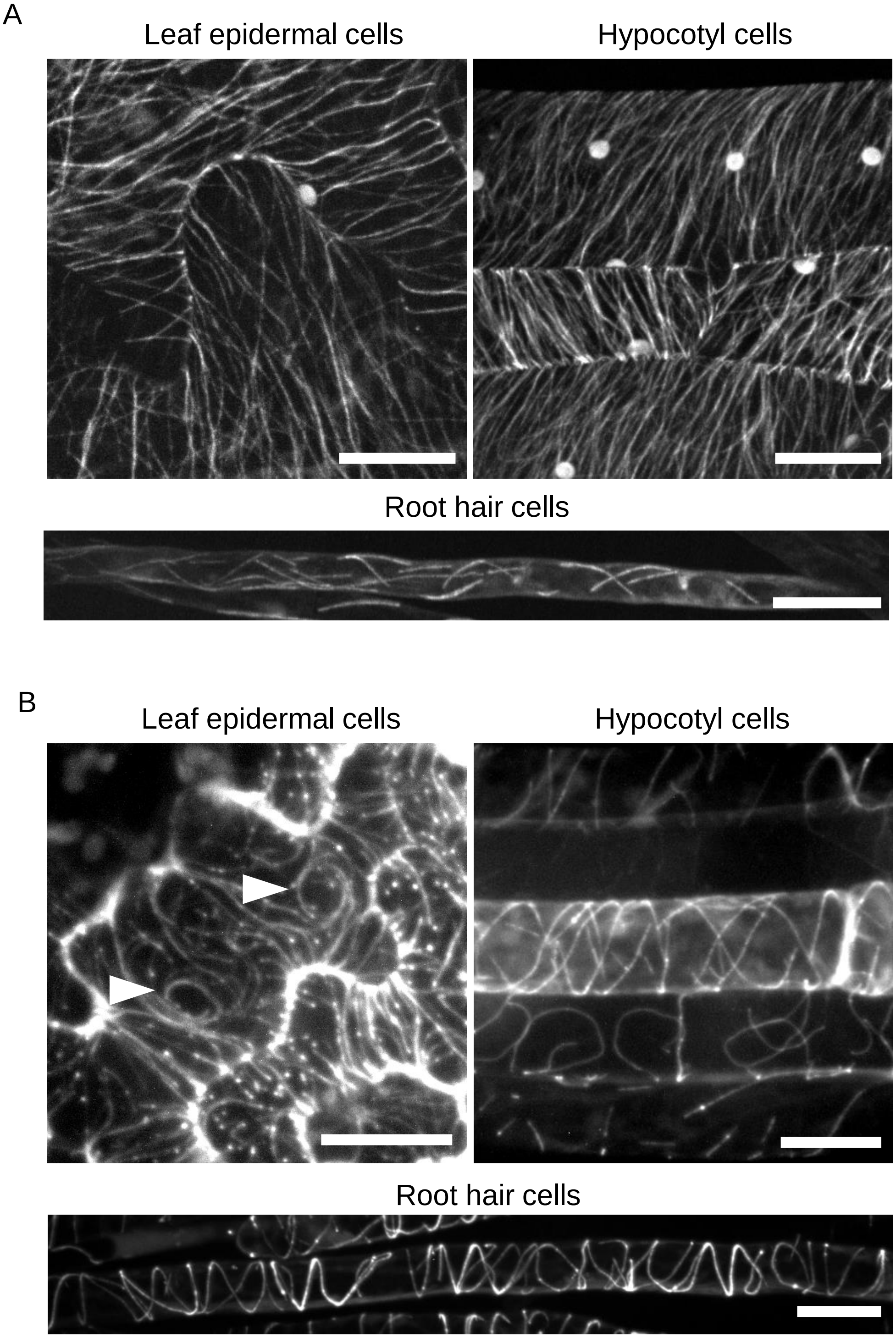

Supplemental Figure 4. Ectopic MAP70-5 expression promotes microtubule bending in planta
(A and B) TUB6 (*pUBQ10:EYFP-TUB6*) (A) and MAP70-5 (*pLexA:EYFP-MAP70-5*) (B) in leaf epidermal cells, hypocotyl epidermal cells, and root hairs. Seedlings were observed after 3-day-long treatment with estrogen. Arrowheads indicate circular bundled microtubules.
Scale bars: 20 µm

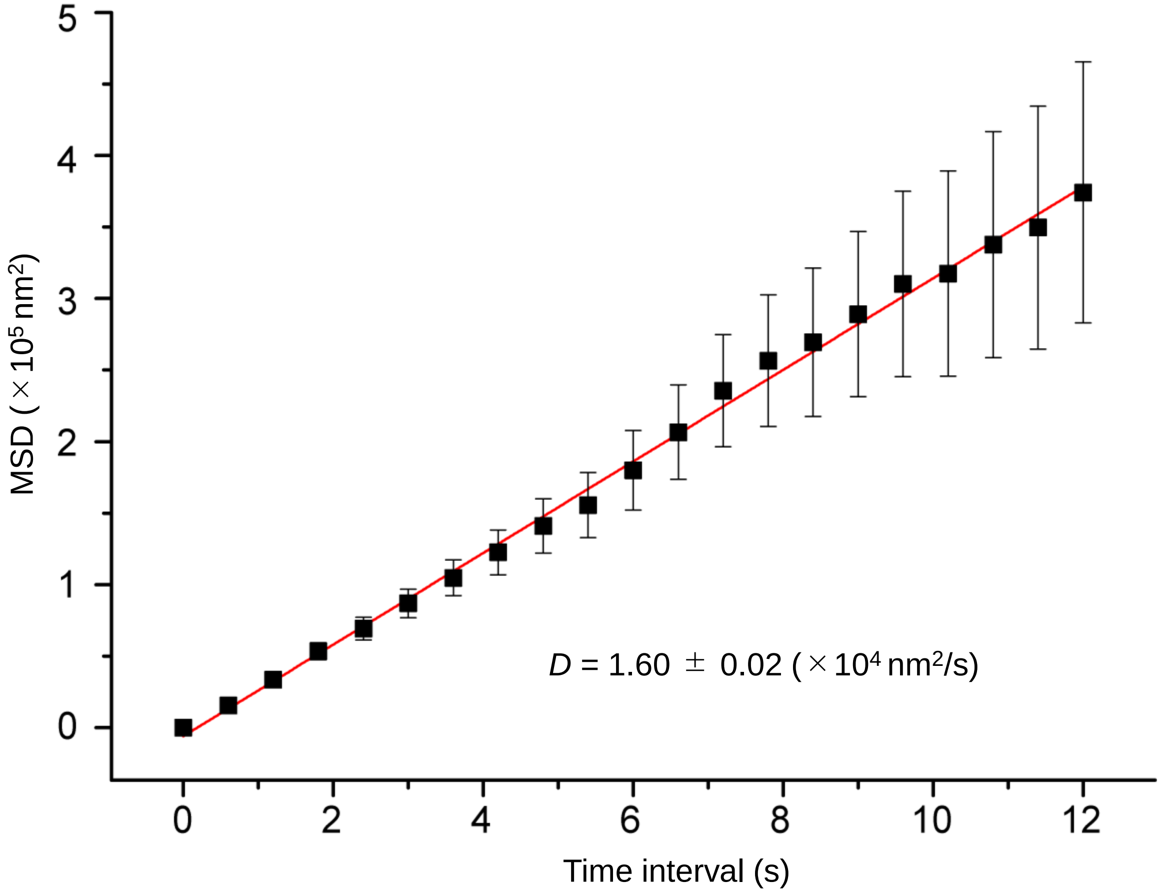

Supplemental Figure 5. MSD analysis of GFP-MAP70-5

Mean Square Displacement (MSD) analysis of the movement of 3 nM GFP-MAP70-5 molecules (n = 73 traces). Bars indicate standard error. Value for diffusion coefficient (*D*) from the linear fit (red line) of the data to MSD = 2*Dt* + offset is indicated.

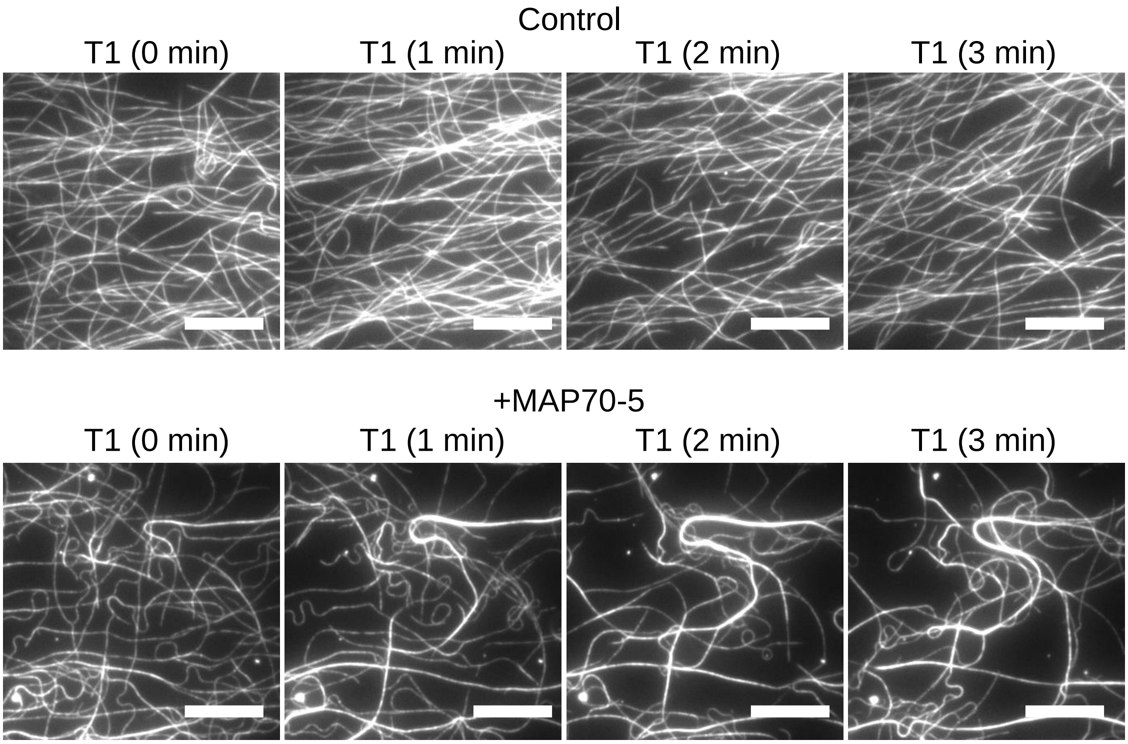

Supplemental Figure 6. Time series of gliding ATTO565-microtubules in the absence (Control) or presence (+MAP70-5) of 2 μM GFP-MAP70-5.

Observation began 10 sec after the addition of buffer (Control) or MAP70-5 (+MAP70-5).

Scale bars: 10 μm

Supplemental Table 1 Primer list

Primer name	Sequence
MAP70-1-CFW	ATTGATGCTGAGATCAAAGCGTTG
MAP70-1-CRV	AAACCAACGCTTTGATCTCAGCAT
MAP70-5-CFW	ATTGAGGAAGTTGGAGGAGAAACT
MAP70-5-CRV	AAACAGTTTCTCCTCCAACTTCCT
MAP70-5-804-inF-R	GCGCCCACCCTTCTACTCATCTTTCCACTC
pEntr_TAG-F	TAGAAGGGTGGGCGCGCCGAC
MAP70-5-805-inF-F	GCCCCCTTCACCATGAACGATAGAGTCATG
pEntr_R	CATGGTGAAGGGGGCGGC
MAP70-5-301F	ACTCACCAACAGGACGATGACTCTC
MAP70-5-300del	GTCCTGTTGGTGAGTCATGGTGAAGGGGGC
MAP70-5-300R	GTAAACCCTCCTTAGTGCTTCTTC
MAP70-5-300-493-F	CTAAGGAGGGTTTACTTGATTGTTGAAGAG
MAP70-5-462R	AGCACGTTCAAGGGCACTTCTC
MAP70-5-462-655-F	GCCCTTGAACGTGCTGGAGGAACAGCCGCC
MAP70-5-654R	TGCCAAGATCGCTTCCTCCAGTTC
MAP70-5-654-805-InF-F	gaagcgatcttggcaAACGATAGAGTCATG
MIDD1-NdeI-inF-R-Long2	GGTGAAGGGGGCGGCCGCGGAGCCTGCTTTTTTGTACAAACTTGTGATGTACAGCTCGTCCATGCC
MIDD1-361-InF-F	GCCGCCCCCTTCACCTCGGAAGATTCAAGAATTG
MIDD1pro-GFPf1	TAAACTCCAAAAATGGTGAGCAAGGGCGAG
MIDD1pro-r1	TTTTGGAGTTTAAAAACCTTTTC
MIDD1-f1	ATGCAGACTCCAAAATC
pEnt-MIDD1-r2	TGGAGTCTGCATGGTGAAGGGGGCGGCC
caccMAP70-5f1	CACCATGACTGCAGCAGAAAACCC
MAP70-5-R	CTGGCAACGTGGATTGGGTATG
MAP70-5pro2kF	CTCCGCGGCCGGGTCAGAATGATGATGGC
proGUS-F	CCGGTATAAGTCAACTGGCG
proGUS-R	TTTGTAGTCTCAACGGCTCCTCC
