# Supplementary material for: Confined-microtubule assembly shapes three-dimensional cell wall structures in xylem vessels
Source: Nat Commun. 2023 Nov 13;14:6987. doi: 10.1038/s41467-023-42487-w (PMC10643555; doi:10.1038/s41467-023-42487-w)
Supplement: Supplementary file 3 — Description of Additional Supplementary Files [file 41467_2023_42487_MOESM3_ESM.pdf]

Description of additional supplementary files

Supplementary Movie 1. EB1b (pXCP1:GFP-EB1b) in metaxylem vessel cell of wild type plant. Images were acquired every 2 sec. The movie shows 5 frames/s. Scale bar: 5 µm.

Supplementary Movie 2. EB1b (pXCP1:GFP-EB1b) in metaxylem vessel cell of map70-1 map70-5 plant. Images were acquired every 2 sec. The movie shows 5 frames/s. Scale bar: 5 µm.

Supplementary Movie 3. Cortical microtubules (pUBQ10:tagRFP-TUB6) in leaf epidermal cells of N. benthamiana expressing GFP-MAP70-5 (pLexA:GFP-MAP70-5). Images were acquired every 15 min. The movie shows 5 frames/s. Scale bar: 10 µm.

Supplementary Movie 4. X-rhodamine-microtubules (red) and GFP-MAP70-5 (green). Microtubules were incubated with 3 nM GFP-MAP70-5. Images were acquired every 5 sec. The movie shows 5 frames/s. Scale bar: 10 µm.

Supplementary Movie 5. ATTO565-microtubules in control buffer. Images were acquired every 5 sec. The movie shows 5 frames/s. Scale bar: 10 µm.

Supplementary Movie 6. ATTO565-microtubules in GFP-MAP70-5-containing buffer. Images were acquired every 5 sec. The movie shows 5 frames/s. Scale bar: 10 µm.

Supplementary Movie 7. Low concentration of ATTO565-microtubules in control buffer. Images were acquired every 5 sec. The movie shows 5 frames/s. Scale bar: 20 µm.

Supplementary Movie 8. Low concentration of ATTO565-microtubules in GFP-MAP70-5- containing buffer. Images were acquired every 5 sec. The movie shows 5 frames/s. Scale bar: 20 µm.

Supplementary Movie 9. ATTO565-microtubules in GFP-MAP70-5-containing buffer. Images were acquired every 5 sec. The movie shows 5 frames/s. Scale bar: 10 µm.

Supplementary Movie 10. A biotinylated X-rhodamine-microtubule tethered to a bead in 1 µM 6xHis-GFP-MAP70-5. Images were acquired every 100 msec. The movie shows 20 frames/s. Scale bar: 5 µm.
